# Feasibility of using software-aided selection of virtual monoenergetic level for optimal image quality of acute necrotising pancreatitis based on dual-energy computed tomography: a preliminary study

**DOI:** 10.1186/s12880-023-01032-3

**Published:** 2023-07-18

**Authors:** Yuan Yuan, Kai Liao, Zixing Huang, Liping Deng, Hehan Tang, Yi Wang, Zheng Ye, Xinyue Chen, Bin Song, Zhenlin Li

**Affiliations:** 1 Department of Radiology, West China Hospital, Sichuan University, No. 37 Guoxue Lane, Chengdu, 610041 Sichuan P.R. China; 2 CT collaboration, Siemens-healthineers, Chengdu, 610041 Sichuan P.R. China

**Keywords:** Acute necrotising pancreatitis, Dual-source computed tomography, Monoenergetic spectrum, Optimal single-energy level imaging for contrast-to-noise ratio, Signal-to-noise ratio

## Abstract

**Objective:**

This study aimed to assess the feasibility of software-aided selection of monoenergetic level for acute necrotising pancreatitis (ANP) depiction compared to other automatic image series generated using dual-energy computed tomography (CT).

**Methods:**

The contrast-enhanced dual-source dual-energy CT images in the portal venous phase of 48 patients with ANP were retrospectively analysed. Contrast-to-noise ratio (CNR) of pancreatic parenchyma-to-necrosis, signal-to-noise ratio (SNR) of the pancreas, image noise, and score of subjective diagnosis were measured, calculated, and compared among the CT images of 100 kV, Sn140 kV, weighted-average 120 kV, and optimal single-energy level for CNR.

**Results:**

CNR of pancreatic parenchyma-to-necrosis in the images of 100 kV, Sn140 kV, weighted-average 120 kV, and the optimal single-energy level for CNR was 5.18 ± 2.39, 3.13 ± 1.35, 5.69 ± 2.35, and 9.99 ± 5.86, respectively; SNR of the pancreas in each group was 6.31 ± 2.77, 4.27 ± 1.56, 7.21 ± 2.69, and 11.83 ± 6.30, respectively; image noise in each group was 18.78 ± 5.20, 17.79 ± 4.63, 13.28 ± 3.13, and 9.31 ± 2.96, respectively; and score of subjective diagnosis in each group was 3.56 ± 0.50, 3.00 ± 0.55, 3.48 ± 0.55, and 3.88 ± 0.33, respectively. The four measurements of the optimal single-energy level for CNR images were significantly different from those of images in the other three groups (*P* < 0.05). CNR of pancreatic parenchyma-to-necrosis, SNR of the pancreas, and score of subjective diagnosis in the images of the optimal single-energy level for CNR were significantly higher, while the image noise was lower than those in the other three groups (all *P* = 0.000).

**Conclusion:**

Optimal single-energy level imaging for CNR of dual-source CT could improve quality of CT images in patients with ANP, enhancing the display of necrosis in the pancreas.

## Introduction

Acute pancreatitis (AP) is a common inflammatory disorder, which is the leading cause of gastrointestinal disorder-associated hospitalisation in the United States and other countries [1]. There is a demand for advanced medical and interventional care since development of severe and/or necrotising pancreatitis is estimated to occur in 20% of the patients [2]. Acute necrotising pancreatitis (ANP) refers to the event of necrosis occurring in either the pancreatic parenchyma, peripancreatic tissues, or both regions [3], leading to complications, such as multiple organ failure [4]. Due to the high incidence and mortality related to pancreatic necrosis, management that involves multidiscipline perspectives (gastroenterologists, surgeons, etc.) is required [5].

Accurate characterisation of local complications, such as pancreatic and peripancreatic fluid or necrosis, the time course of progression, and whether infection is present, will contribute to improved patient stratification, either for clinical care in specialised centers or for clinical investigation reports [6]. Computed tomography (CT) can serve as a principal imaging tool for diagnosing AP and relevant complications along with prognostic prediction, while contrast-enhanced CT (CECT) can be applied to diagnose and quantify pancreatic necrosis [7]. Furthermore, CECT is recommended at the beginning of the advanced stage of the disease to identify increased risk of poor prognosis in suspected or confirmed ANP patients [8]. Modern cross-sectional imaging with CECT scanning has been reported as the gold standard for diagnosis and substantiation of infected necrosis, which alters the therapeutic recommendations from conservative treatments to interventional and ultimately surgical therapies [9].

With the introduction of a new dual-source CT system, the dual-energy CT (DECT) technique can be utilised in the imaging of the abdomen, providing potential clinical applications for pancreatic evaluation [10]. With the widespread acceptance of this technique, DECT shows great promise for pancreatic imaging and eventually for pancreatitis-related confounding factors, encompassing necrosis, blood vessel complications, and pancreatic/peripancreatic collections [11]. A preliminary study has highlighted the value of DECT in assessing the complexity of pancreatic/peripancreatic collections as well as residual parenchyma enhancement [12]. DECT is superior to conventional single-energy CT (SECT) as it can use energy spectrum information to compensate for insufficient image quality, deficient contrast bolus, and metallic artefacts through virtual energetic technique. These improve the CT-based evaluation for gastrointestinal disorders [13]. However, the optimal monoenergetic level has not been determined for each diagnostic task by consensus or guidelines. Moreover, questions have also arisen on how to reduce operator variation in the selection of monoenergetic level for this application. To address these, we used software-guided selection of monoenergetic level and compared this with image series that were automatically generated in terms of image quality for ANP depiction. In the current work, dual-source DECT scans were conducted for ANP patients to evaluate the value of optimal iodine contrast-to-noise ratio (CNR) single-energy images with dual-source CT single-energy spectrum technology in optimising ANP image quality.

## Patients and methods

### Ethics statement

This study was approved by the institutional review board of the West China Hospital, Sichuan University (No. 2,019,117). All participants were informed of the specific details of the study and signed the informed consents before enrollment.

### Patient population

Forty-eight patients who were hospitalised in our institution from March, 2015 to January, 2016, diagnosed with ANP through clinical data, laboratory tests, and imaging and underwent abdominal contrast-enhanced dual-source DECT examinations were retrospectively analysed. These 48 patients (24 men and 24 women) were aged between 19 and 73 years, with an average age of 46 years.

### Examinations

#### Preparation before scanning

Metallic objects, high-density ornaments, waists, belts, and topical medications were removed from the abdomen of patients to minimise the generation of beam hardening artefacts. Before the examination, all patients signed the informed consents for contrast agents and underwent breath-holding training. During the examination, the patients were instructed to cooperate with breath-holding according to the voice prompts to avoid motion artefacts from breathing and keep the amplitude of each breath as consistent as possible to avoid leaky layers and repeated scanning.

### CT scanning regimen

The second-generation dual-source CT (Somatom Definition Flash, Siemens Healthcare, Germany) was used to perform abdominal contrast-enhanced DECT scans. The sequence included a nonenhanced scan and dual-phase enhanced scan (arterial and portal venous phases). The range for nonenhanced and venous phase scans were from the dome of the diaphragm to the pelvic cavity, while the range for arterial phase scans were from the dome of the diaphragm to the uncinate process of the pancreas. The 120-kVp SECT was utilised to acquire both nonenhanced and arterial phase scans of the abdomen with the reference tube current set at 210 mAs. A DECT protocol with tube voltage of 100-kV and 140 kV was used for the venous phase. Reference tube current was set to 300 mAs and 232 mAs, respectively. The 140-kVp was equipped with an additional tin filter. Further settings were adjusted for 120-kVp SECT (pitch, 0.7; rotation time, 0.5 s; and collimation, 128 × 0.6 mm) and DECT (pitch, 0.7; rotation time, 0.5 s; and collimation, 32 × 0.6 mm). During the acquisition, both angular and longitudinal dose was modulated by the automatic exposure control software (Care Dose 4D, Siemens Healthcare). Images were reconstructed with a medium smooth kernel (B30f), 1.5-mm slice thickness, and 1.0-mm slice increments. The contrast, Omnipaque (iohexol injection, Shanghai GE Pharmaceutical Co., Ltd., Shanghai, China, 100 mL/bottle, 300 mgI/mL, nonionic contrast), was utilised for dual-phase enhanced scanning, with a total contrast agent of 1.5 mL/kg body weight. High pressure injection was performed through forearm vein mass injection at the rate of 2.5–3.0 mL/s, and the arterial and portal phase images were collected at 35 and 70 s after injection of contrast agents using the high pressure injector (Stellant, Medrad, Inianola, USA) [14]. Images of 100 kV, Sn140kV, and 50% weighted-average of both (120 kV) were obtained through scanning [15].

### Image data measurement and quality score

**Fig. 1:**
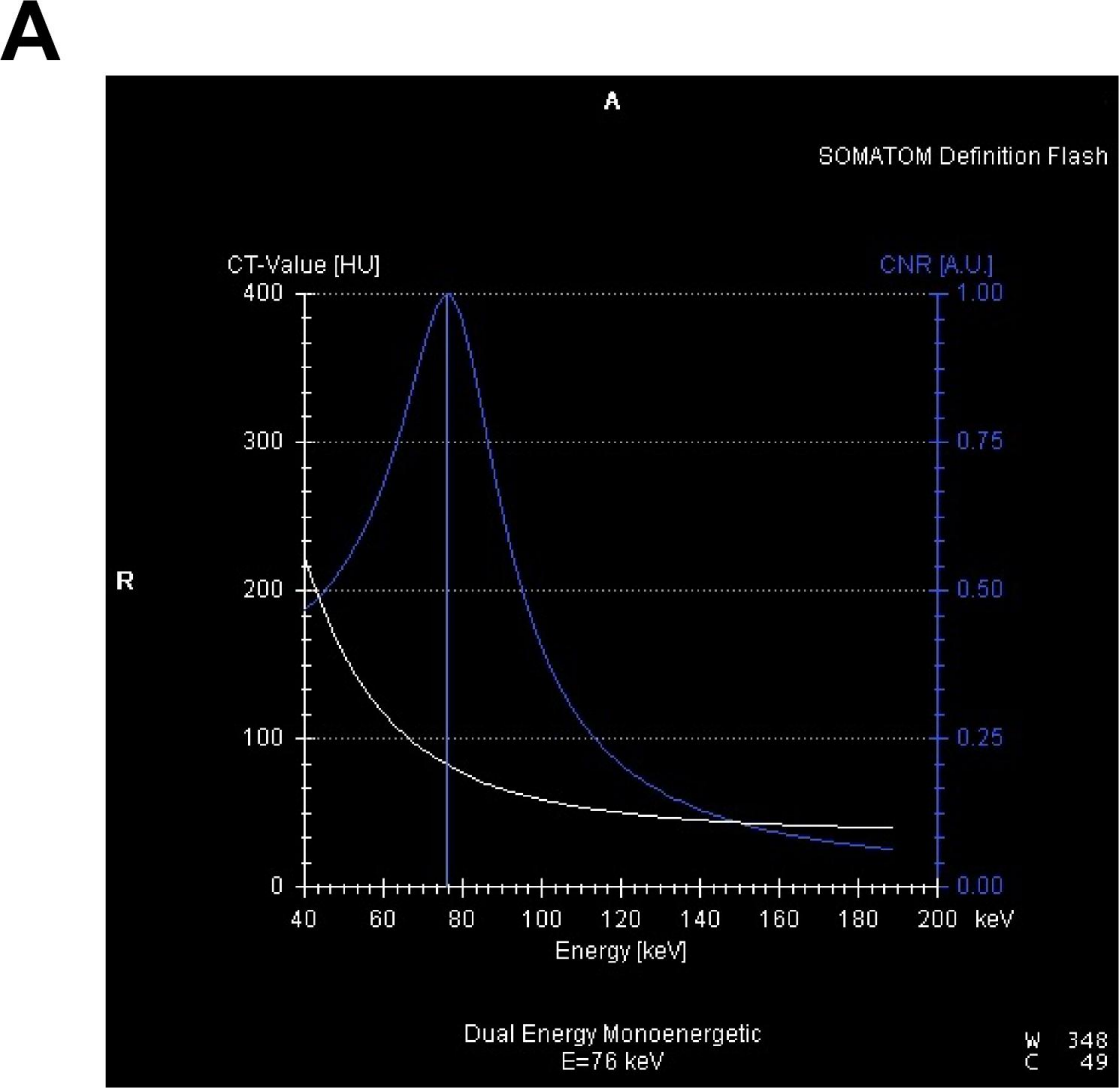
The curve of iodine contrast-noise-ratio which was changed with the energy level

Three sets of data were directly obtained from the portal phase dual-energy scan: 100 kV, Sn140 kV, and weighted-average 120 kV data. All were reconstructed as images with layer thickness of 1.5 mm and interlayer spacing of 1.0 mm. Additionally, 100 kV and Sn140 kV data were transmitted through a picture archiving and communication system to the Dual-Energy software of the Siemens Syngo MMWP VE36A Workstation, and the pancreatic parenchyma was delineated using the spectral information option in the monoenergetic program, obtaining the curve of iodine CNR against the energy level (keV) (Fig. 1). The image data for the CNR single-energy value was extracted. Next, the obtained 100 kV, Sn140 kV, weighted-average 120 kV, and the optimal CNR single-energy images (a total of four sets of images) were transferred into the Viewing window to manually delineate the regions of interest (ROI). On the 100 kV images, the ROI of pancreatic necrotic foci and of the adjacent pancreatic parenchyma without apparent necrosis were delineated, and the CT values in the ROI were measured. The CT value measurements of the other three groups of images were performed at the same level, the same site and in the same ROI. The pancreatic necrosis focal CT values (N values) and adjacent pancreatic parenchymal CT values (P values) were recorded separately for each group of images, followed by measurement of the standard deviation (SD value) of the fat CT value in the uniform fat area of the subcutaneous abdominal wall. The signal-to-noise ratio (SNR) of the pancreas was calculated using the pancreatic CT value/SD value, and the CNR of pancreatic parenchyma-necrosis was calculated with (P-N) value/SD value.

The images obtained were subjectively evaluated by two radiologists each with over 10 years of experience of abdominal imaging diagnosis, and the four groups of images were graded with 1–4 points according to the clarity of the pancreatic necrosis focus and diagnostic information provided by the images. The score standards were as follows [16]: 4, very clear pancreatic necrosis focus, with the image providing sufficient diagnostic information; 3, relatively clear pancreatic necrosis focus, with the image providing enough diagnostic information; 2, unclear pancreatic necrosis focus, with the image providing insufficient diagnostic information; and 1, no pancreatic necrosis focus, with the image providing no diagnostic information. A score of image quality ≥ 3 points was considered to meet the diagnostic requirement. Any inconsistencies were determined through consultation.

### Radiation dose assessment

The volume CT dose index (CTDI_vol_) and dose-length product (DLP) of each patient were recorded separately. Effective dose (ED) was calculated with the following formula: ED = DLP × conversion coefficient (0.015 mSV/mGy·cm).

### Statistical analysis

The measurement data were represented by mean value ± SD, and counting data were expressed in percentages or rates. Analysis was performed using the Statistical Package for the Social Sciences 22.0 statistical software. The SNR of the pancreas, CNR of pancreatic parenchyma-necrosis, and image noise of the four groups were compared using repeated measures one-way analysis of variance (ANOVA). The multiple comparisons were analysed using the Student–Newman–Keuls (SNK) method. The score of subjective diagnosis was compared using a nonparametric test (Friedman rank sum test) of multiple independent samples, and the consistency of observers was analysed using a kappa test (a kappa value of 0, no consistency; ≤ 0.40, poor consistency; 0.40–0.75, medium consistency; and ≥ 0.75, good consistency). The test level was α = 0.05, and *P* < 0.05 was statistically significant.

## Results

### Optimal CNR single-energy value

**Fig. 2:**
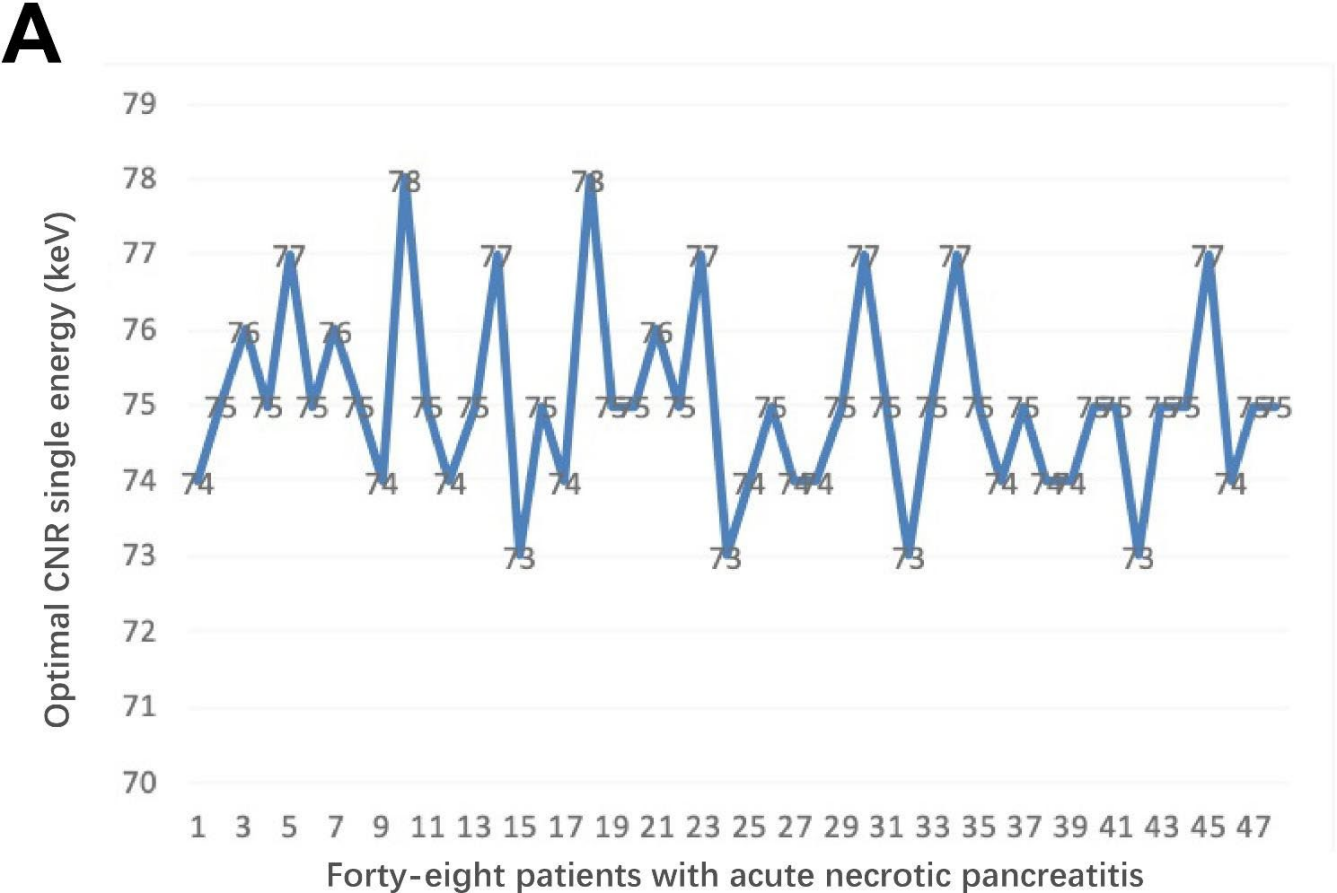
The value of the optimal single-energy level for CNR of 48 patients with acute necrotising pancreatitis

As shown in Fig. 2, the images of 48 patients were analysed using single-energy spectra to obtain their respective CNR curves against keV values. The keV corresponding to the curve peak was the optimal CNR single-energy value. The average optimal CNR single-energy value for the 48 patients was 75.04 ± 1.24 keV, ranging from 73 to 78 keV.

### CNR of pancreatic parenchyma-necrosis

**Fig. 3:**
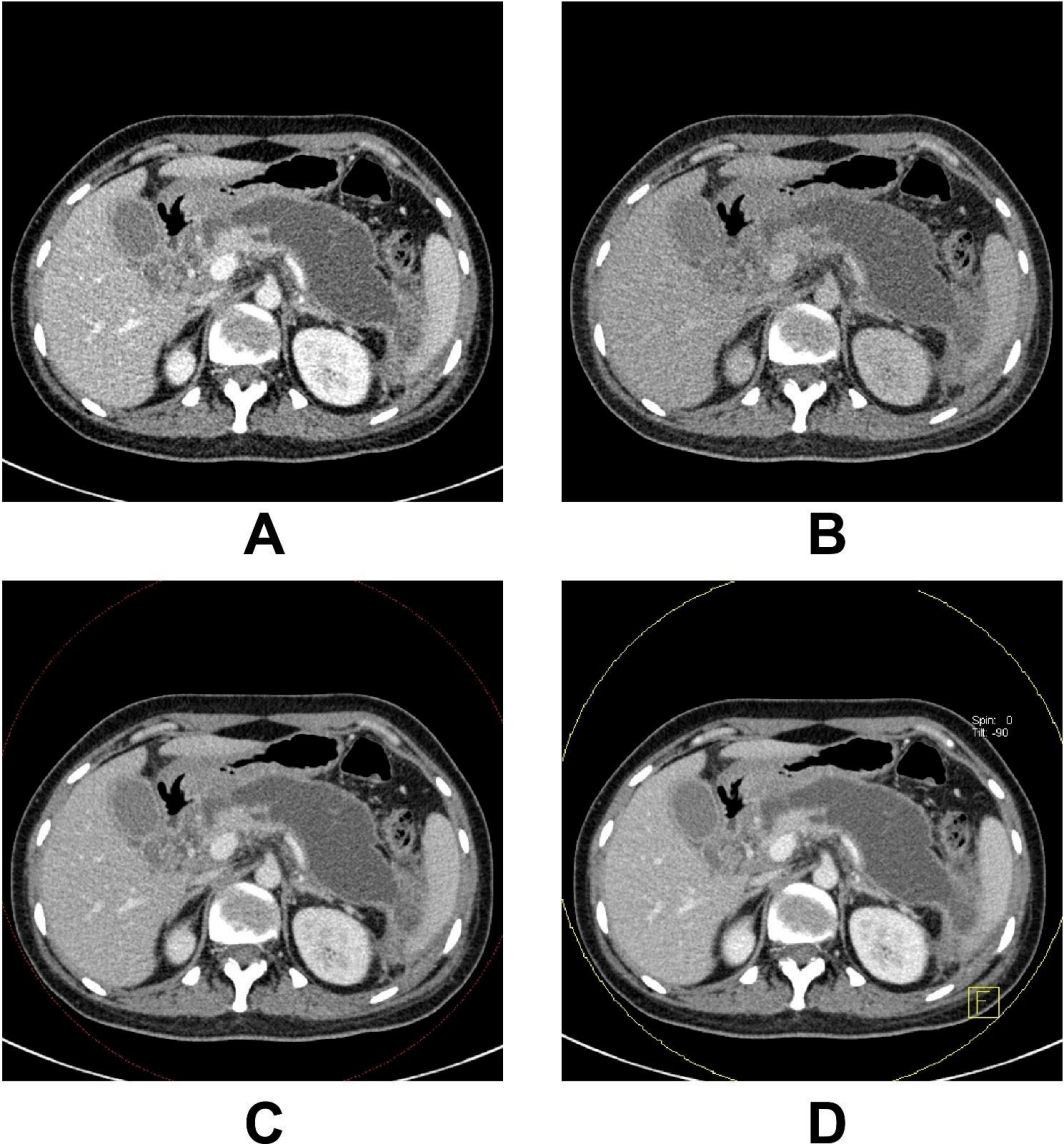
The images of 100 kV (**A**), Sn140 kV (**B**), weighted-average 120 kV (**C**) and the optimal single-energy level for CNR (**D**) acquired from contrast-enhanced dual-source dual-energy CT in portal venous phase of acute necrotising pancreatitis

Results of pancreatic parenchyma-necrosis CNR are shown in Table 1; Fig. 3. Analysis using repeated measures one-way ANOVA showed there were significant differences for the CNR of pancreatic parenchyma-necrosis (*P* < 0.05) in the images of 100 kV, Sn140 kV, weighted-average 120 kV, and the optimal single-energy level for CNR. The SNK test showed no significant difference between the 100 kV and weighted-average 120 kV images, while comparisons among the other three groups of images exhibited significant differences (*P* < 0.05). Among them, the largest CNR for pancreatic parenchyma-necrosis was found in the optimal single-energy level for CNR images, followed by the images of weighted-average 120 kV, 100 kV, and Sn140 kV, respectively.

**Table 1:** Measurement results in the images of 100 kV, Sn140 kV, weighted-average 120 kV, and the optimal single energy level for CNR acquired from contrast-enhanced dual-source dual-energy CT in portal venous phase of acute necrotizing pancreatitis

	n	CNR of pancreatic parenchyma-to-necrosis	SNR of pancreas	Image noise	Score of subjective diagnosis
100 kV	48	5.18 ± 2.39	6.31 ± 2.77	18.78 ± 5.20	3.56 ± 0.50
Sn140 kV	48	3.13 ± 1.35	4.27 ± 1.56	17.79 ± 4.63	3.00 ± 0.55
Weighted- average 120 kV	48	5.69 ± 2.35	7.21 ± 2.69	13.28 ± 3.13	3.48 ± 0.55
The optimal single energy level for CNR	48	9.99 ± 5.86	11.83 ± 6.30	9.31 ± 2.96	3.88 ± 0.33
*F*		33.589	34.300	54.583	26.261
*P*		0.000	0.000	0.000	0.000

Note: CNR, contrast-to-noise ratio; SNR, signal-to-noise ratio

### SNR of the pancreas

Similarly, there were significant differences for the SNR of the pancreas analysed using repeated measures one-way ANOVA (*P* < 0.05) in the images of 100 kV, Sn140 kV, weighted-average 120 kV, and the optimal single-energy level for CNR. The SNK test suggested no significant difference between the images of 100 kV and weighted-average 120 kV, while substantial differences were observed in the comparisons among the other three groups of images (*P* < 0.05). Among them, the largest SNR of the pancreas was found in the optimal single-energy level for CNR images, followed by the images of weighted-average 120 kV, 100 kV and Sn140 kV, respectively (Table 1; Fig. 3).

### Image noise

As displayed in Table 1; Fig. 3, the image noise exhibited significant differences in the images of 100 kV, Sn140 kV, weighted-average 120 kV, and the optimal single-energy level for CNR, analysed using repeated measures one-way ANOVA (*P* < 0.05). Based on the SNK test, no significant differences were found between the images of 100 kV and Sn140 kV, while significant differences were found in the comparisons among the other three groups of images (*P* < 0.05). Among them, the smallest image noise was witnessed in the optimal single-energy level for CNR images, followed by the images of weighted-average 120 kV, Sn140 kV, and 100 kV, respectively.

### Score of subjective diagnosis

Findings of the score of subjective diagnosis are shown in Table 1; Fig. 3. The scoring differences among four groups were analysed using the non-parametric equivalent Friedman rank sum test in repeated measures one-way ANOVA test. There were significant differences among the four groups (*P* < 0.05). Conover’s all-pairs test was used to compare the differences between the 4 groups in pairs, and bonferroni was used to correct the *P* values of these tests. It was found that there were no significant differences in the scores of mixed 120 kV and 100 kV, while the comparisons among the other three groups of images exhibited significant differences (*P* < 0.05). Among them, the largest score of subjective diagnosis was found in the optimal single-energy level for CNR images, followed by the images of 100 kV, weighted-average 120 kV, and Sn140 kV, respectively.

### Radiation dose comparison

Finally, we recorded the radiation dose. The CTDI_vol_ values of nonenhanced scan and dual-phase enhanced scan (arterial and portal venous phases) in the three phases were 9.31 ± 1.67 mGy, 8.78 ± 1.58 mGy, and 14.52 ± 2.61 mGy, respectively. The DLP values in the three phases were 493.39 ± 130.09 mGy·cm, 244.51 ± 64.47 mGy·cm, and 775.51 ± 204.07 mGy·cm, respectively. According to the conversion coefficient, the ED values were calculated as 7.40 ± 1.95 mSv, 3.67 ± 0.97 mSv, and 11.63 ± 3.06 mSv, respectively.

## Discussion

Pancreatitis is an inflammatory disorder that involves autodigestive injury of the pancreatic tissue caused by the premature activation of digestive enzymes in the pancreas, in particular pathologic activation of trypsinogen during early stages [17]. AP can be divided into interstitial oedematous pancreatitis and ANP based on different morphological characteristics [6]. Pancreatic necrosis is related to organ failure and a rise in mortality risk [18]. CT serves as the most frequently used imaging test for the diagnosis of AP and differentiation between acute interstitial pancreatitis and ANP and moreover a useful detector of parenchymal necrosis approximately 72 h after appearance of symptoms [19, 20]. Therefore, early detection of AP and accurate grading of disease severity may aid in timely and appropriate treatment, and early diagnosis of ANP can potentially contribute to developing specialised clinical therapeutic regimens.

CECT images show the presence of necrosis as single or multiple regions of nonenhancing pancreatic parenchyma in ANP patients [7]. Additionally, multislice CECT scans are beneficial for identifying the complications of AP due to its value in disease diagnosis and severity grading [21]. Recently, the advance of DECT with iodine quantification allows differentiation of normal pancreatic parenchyma from inflammatory pancreatic parenchyma and exerts superior sensitivity for diagnosing early AP compared to standard imaging methods [22]. Dual-energy spectral CT iodine substance analysis better detects the presence of pancreatic microcirculation injuries in AP and offers material decomposition image analysis for assessing the severity of AP [23]. DECT has applications for oncologic and nononcologic pancreatic imaging. It achieves dual-energy spectra scans by acquiring images with two different tube voltages (i.e., 80 and 140 kVp). Due to energy-dependent photoelectric effects and different K-edges for different elements, DECT can differentiate structures of similar densities and diverse elemental compositions (such as calcium and iodine) [24]. Contrast-enhanced dual-source and DECT scans at a low-energy (80 kVp) can address differences concerning attenuation between necrotic loci and normal pancreas, thereby improving CNR and subjective evaluation of necrosis [12]. In this study, we found the optimal single-energy value for CNR in 48 patients to be on average 75.04 ± 1.24 keV, ranging from 73 keV to 78 keV.

In addition, the optimal single-energy value corresponding to the CNR peak can be determined through the iodine CNR curve. The images from this single-energy value can improve the iodine contrast and CNR, thus providing optimised low-noise and high-contrast images and improving diagnostic performance of the lesions. A recent study has documented a two-fold attenuation difference between normal and inflamed parenchyma and noticeable increases in the SNR and CNR values of single-energy images versus conventional images [25]. The results in this study have revealed that the optimal single energy for CNR images had the highest CNR value of pancreatic parenchyma-to-necrosis, pancreatic SNR, and subjective score compared with those of the 100 kV, Sn140kV, and weighted-average 120 kV mixed-energy images of the other three groups, acquired using dual-energy direct CT scanning. Additionally, optimal CNR single-energy images showed the least noise. These results suggest that compared with the mixed-energy images from dual-energy direct CT scans, the optimal CNR single-energy images can reduce image noise and elevate the pancreatic SNR and CNR values of pancreatic parenchyma-to-necrosis. Therefore, this technique can improve the overall quality of CT images and subjective diagnoses for pancreatic necrosis, providing a powerful imaging tool for its early detection.

This study had some limitations. Firstly, the sample size was small. Therefore, trials with an expanded sample size are necessary in the future to increase confidence in these results. Secondly, the single-energy spectrum images within the range of 40–190 keV were not analysed. In the future, the single-energy spectrum images at different keV values should be compared and analysed to acquire images of better quality.

In summary, compared with 100 kV, Sn140 kV, and weighted-average 120 kV images, the optimal single-energy CNR images can reduce image noise, increase SNR of the pancreatic tissues and CNR of the pancreatic parenchyma-to-necrosis, and improve the subjective diagnosis for pancreatic necrosis. The overall quality of the images is thereby improved, which is conducive to the identification of necrotic loci in ANP.

## Data Availability

The datasets used or analyzed during the current study are available from the corresponding author on reasonable request.
